# Retinoblastoma in Adults: Clinical Features, Gene Mutations and Treatment Outcomes: *A Study of Six Cases*

**DOI:** 10.3389/fonc.2022.835965

**Published:** 2022-08-02

**Authors:** Nan Zhou, Lihong Yang, Xiaolin Xu, Yueming Liu, Wenbin Wei

**Affiliations:** Beijing Tongren Eye Center, Beijing Key Laboratory of Intraocular Tumor Diagnosis and Treatment, Medical Artificial Intelligence Research and Verification Laboratory of the Ministry of Industry and Information Technology, Beijing Tongren Hospital, Capital Medical University, Beijing, China

**Keywords:** retinoblastoma, onset in adult, clinical features, genetic analysis, treatment outcomes

## Abstract

**Purpose:**

To report six Asian adult patients with retinoblastoma (RB).

**Design:**

Retrospective and observational small case series.

**Participants:**

Six patients with a white dome-shaped tumor of the retina were evaluated from May 10, 1995, to September 10, 2021.

**Main Outcome Measures:**

Initial tumor and associated fundus features, pathology, gene mutation, treatment, tumor course on follow-up, and salvage globe outcome.

**Results:**

The six affected Asian patients consisted of three men and three women. The mean age at the time of diagnosis was 36.5 years (median: 31 years, range: 20-55 years). All patients were unilateral. In all cases, the tumors were white, dome-shaped, with full-thickness retinal involvement, and mushroom-like protrusions into the vitreous cavity. The mean tumor thickness measured by ultrasonography was 4.5 mm (median: 3.2 mm, range: 3.2-6.8 mm). Associated characteristic symptoms included dilated retinal feeding artery and draining vein (100%), surrounding subretinal infiltration (83%), exudative retinal detachment (83%), and vitreous seeds (67%). Local tumor resection was performed in three patients, I-125 plaque brachytherapy combined with transpupillary thermotherapy (TTT) and intravitreous injection of melphalan (combination treatment) in one patient, I-125 plaque brachytherapy in two patients, and enucleation in one (20%) patient. RB1 gene testing was carried out on four patients and pathological diagnosis on five patients. Genetic analysis revealed that the RB1 mutation was a mosaic c.709dupG (p.Glu237GlyfsTer4) duplication in one patient, a mosaic c.763C>T(p.Arg255Ter) mutation in another patient, while the remaining two patients were RB1 negative. At the end of the follow-up, none of the patients had developed tumor-related metastasis or died. The findings were consistent in all patients who had an adequate follow-up. This study focused on this rare lesion to distinguish it from other intraocular white lesions in adults, including choroidal osteoma, vitreoretinal lymphoma, and retinal capillary hemangioma, all of which are different clinical entities.

**Conclusion:**

In adults, RB is typically a white, full-thickness retinal mass that is unilateral, often combining with retinal feeding vessels, subretinal infiltration, and vitreous seeds. Genetic studies on adult-onset RB are essential and still require elucidation. Despite RB being a malignant tumor, patient survival was minimally affected.

## Introduction

Retinoblastoma (RB), a tumor originating from the sensory retina, is the most common primary malignant intraocular tumor in children, with an incidence of one case per 15,000-20,000 live births (1). Approximately 90% of children with RB are diagnosed between birth and five years old, and the tumor has been associated with the RB1 mutation (2).

The occurrence of RB in adults is uncommon, and there is limited published literature on the onset of RB in adults. In 1919, Maghy initially first reported a 20-year-old Caucasian female with bilateral RB (3). Since then, studies on this demographically rare variety of RB have been progressively increasing (4–6). There are less than 30 cases of RB in patients above the age of 20 at the time of diagnosis, with the oldest patient being a 74-year-old man (5). However, because most of them were isolated cases, there was a lack of clinical features and genetic studies on adult-onset RB patients.

The presentation of adult-onset RB can be quite different compared to its pediatric counterpart. Due to its atypical clinical symptoms and delayed diagnosis, elderly RB patients have typically been managed with enucleation. It is important to note the clinical characteristic differences between childhood and adult-onset RB, especially distinguish it from other intraocular white lesions in adults. In this study, we describe the clinical features, treatment outcomes, and review the literature on adult-onset RB based on our experience with six patients.

## Case Reports

A summary of the clinical features, gene mutations, ultrasonographic features, and treatment outcomes of all six cases are provided in **Table 1**.

**Table 1:** Active retinoblastoma in adults: a study of 6 Cases.

Patient	Age/Gender, Years	Laterality/Tumor Location	Tumor-size (mm)	Gene Mutation	IRCB/IRSS	Primary Treatment	Globe Salvage	Outcome	Final BCVA	Follow-up Duration, Months
1	20/F	Unilateral/Intraocular	5.2×3.9×5.9	mosaic c.709dupG (p.Glu237GlyfsTer4) duplication	ICRB-C	Combination Therapy-TTT (3 times), IV-Melphalan	Yes	Alive	NLP	36
2	24/M	Unilateral/Intraocular	5.0×4.3×6.8	mosaic c.763C>T(p.Arg255Ter) mutation in 1	ICRB-D	I-125 plaque brachytherapy; ultimately enucleation	No	Alive	–	46
3	45/F	Unilateral/Intraocular	6.2×4.5×3.9	–	ICRB-C	Local resection	Yes	Alive	20/100	180
4	55/M	Unilateral/Intraocular	5.8×3.9×3.8	–	ICRB-C	Local resection	Yes	Alive	20/200	120
5	38/F	Unilateral/Intraocular	6.1×5.5×6.0	–	ICRB-C	Local resection	Yes	Alive	20/200	72
6	24/F	Unilateral/Intraocular	13.7×4.8×4.7	RB 1 mutation negative	ICRB-D	I-125 plaque brachytherapy-	Yes	Alive	20/40	3

F, Female; M, Male; TTT, transpupillary thermotherapy; BCVA, best corrected visual acuity; IV-Melphalan, intravitreous injection of melphalan; ICRB, International Classification of Retinoblastoma; IRSS, International Retinoblastoma Staging System.

### Patient 1

**Figure 1:**
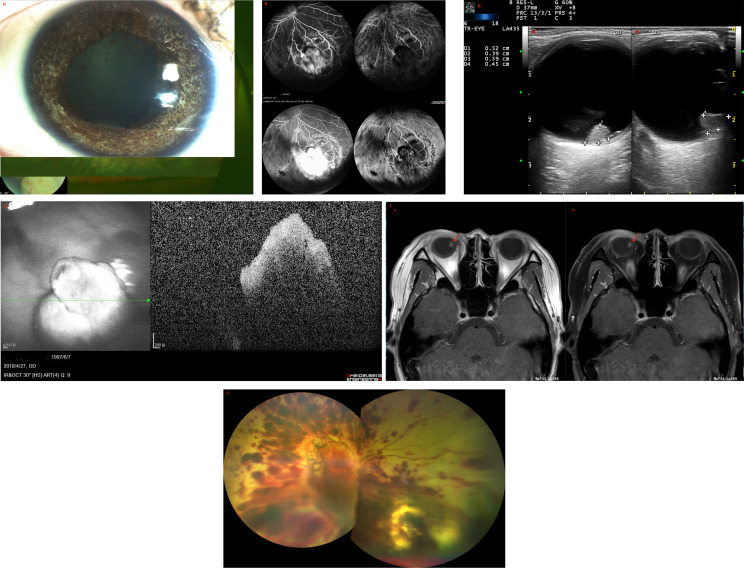
**(A)** Fundus examination of the right eye of patient 1 revealed a large white dome-shaped retinal tumor in the inferonasal quadrant with feeding vessels and a few vitreous cellularities. **(B)** On fundus fluorescein angiography (FA), multiple areas of the tumor displayed mottled hyperfluorescence in the early phase and hyperfluorescence with intense leakage in the late phase. On indocyanine green angiography (ICGA), the tumor showed hypofluorescence during all stages. **(C)** Color Doppler imaging (CDI) revealed a pedunculated mass with inconsistent reflectivity of moderate-intensity and no choroidal excavation, as well as arterial blood signals in the tumor. The size of the elevated lesion was 5.2 × 3.9 × 5.9 mm^3^. **(D)** Optical coherence tomography (OCT) revealed that the tumor had a sloped dome-shaped elevation, with a hyperreflective anterior surface and vitreous seeds, and normal macular fovea. **(E)** MRI revealed that the tumor was far from the optic nerve in the right globe, demonstrating a slightly higher hyperintensity (arrow) than vitreous in **(a)** axial T1-weighted MRI, hypointensity (arrow) in **(b)** axial T2-weighted MRI, and moderate enhancement of the tumor in axial contrast-enhanced T1-weighted, fat-saturated MRI. **(F)** At the last follow-up 15 months after the last combination treatment, the retinoblastoma demonstrated complete regression into a partially calcified scar. **(G)** One day after IV-Melphalan, intravitreal melphalan-hemorrhagic retinopathy toxicity occurred. **(H)** The patient showed signs of iris atrophy and eventually developed phthisis bulbi.

A 20-year-old female was referred to our clinic after experiencing floaters in her right eye for two weeks. The patient had no significant medical history. Upon examination, her visual acuity was 20/50 in the right eye and 20/20 in the left eye. The intraocular pressure (IOP) in the right and left eyes was 12 mmHg and 15 mmHg, respectively. A slit-lamp examination revealed that the anterior segment of both eyes was normal. A dilated fundus examination of the right eye detected a white mass with feeding vessels located in the inferonasal peripheral fundus, which was surrounded by a few vitreous cellularities (**Figure 1A**). The condition of the left eye was normal. Fundus fluorescein angiography (FA) of the neoplasm showed multiple areas of mottled hyperfluorescence in the early stages, followed by obvious staining in the late stages. In contrast, the indocyanine green angiography (ICGA) showed hypofluorescence at all stages (**Figure 1B**). The patient underwent color Doppler imaging (CDI), which revealed a pedunculated mass with moderately inconsistent reflectivity and no choroidal excavation, as well as arterial blood signals in the tumor and no obvious calcification (**Figure 1C**). The size of the elevated lesion was 5.2 × 3.9 × 5.9 mm^3^. Optical coherence tomography (OCT) revealed that the tumor had a sloped dome-shaped elevation with a hyperreflective anterior surface and vitreous seeds, as well as normal fovea horizontally and vertically (**Figure 1D**). MRI showed the right globe with the tumor located far from the optic nerve, demonstrating a slight hyperintensity (arrow) than vitreous in axial T1-weighted MRI, hypointensity (arrow) in the axial T2-weighted MRI, and moderate enhancement of the tumor in the axial contrast-enhanced T1-weighted, fat-saturated MRI (**Figure 1E**). According to genetic analysis, the RB1 gene variant identified in the patient was a mosaic c.709dupG (p.Glu237GlyfsTer4) duplication, which was estimated to be present in approximately 10% of the patient’s blood leukocytes. Targeted PCR-NGS was used to validate the presence of this mosaic variant. The results pointed to RB, which confirmed to the diagnosis. No tumor metastasis was found with 18F-FDG PET/CT. The RB gene test results of the patient’s first-degree relatives were negative.

Combination treatment was then performed. The patient underwent I-125 plaque brachytherapy and three times of transpupillary thermotherapy (TTT) treatments, with a four-week interval between the first and second treatments and an eight-week interval between the second and third treatments. At the last follow-up, which was 30 months following initial presentation and 15 months since last treatment, the RB completely regressed into a partially calcified scar, with complete resolution of intravitreal seeds and no evidence of tumor recurrence (**Figure 1F**). The patient’s visual acuity improved to 20/33 in the right eye and there were no adverse effects during the entire treatment.

Three years after the combination therapy, the recurrence of vitreous seeds was examined with an ophthalmoscope, and the patient was administered intravitreal injection of melphalan (30 ug). After one day of IV-Melphalan, the patient’s BCVA dropped to LP, her IOP was 7 mmHg, and fundus examination revealed that the vitreous seeds had disappeared. However, exudative retinal detachment, choroidal detachment, and preretinal hemorrhage had occurred (**Figure 1G**), which were due to toxicity of intravitreal melphalan-hemorrhagic retinopathy. The patient was given oral prednisone (70 mg/per day) and topical triamcinolone acetonide periocular injection twice, but her condition did not improve. The patient ultimately developed phthisis bulbi (**Figure 1H**) but refused to be enucleated.

### Patient 2

**Figure 2:**
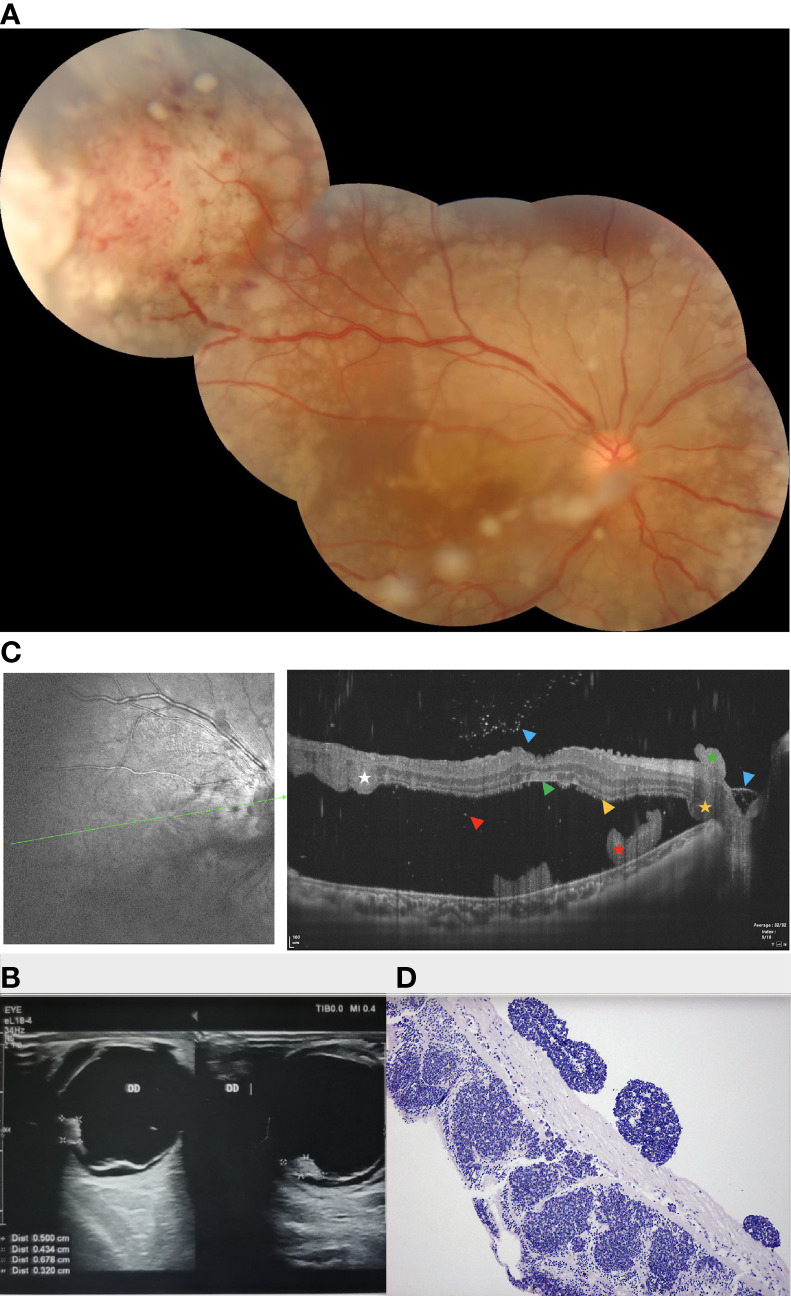
**(A)** Fundus examination of patient 2 revealed a circumscribed, nodular, white lesion of the retina located in the peripheral quadrant, which was associated with tortious feeding vessels and subretinal yellow-white deposits. **(B)** The lesion was 4.3 mm thick and its largest basal diameter was 6.8 mm, as measured by CDI. **(C)** SS-OCT revealed multifocal, punctate, or spot subretinal lesions with medium- to hyper-reflectivity involving the lamina cribrosa and the surrounding of the optic nerve, as well as the spot lesions corresponding to subretinal tumor cells in pathological findings. (Green Triangle: superficial retinal detachment; Red Pentagram: tumor cell cluster; Yellow triangle: damaged photoreceptor cell layer and suspended tumor cells; Red triangle: discrete subretinal tumor cells; Blue triangle: tumor cells are implanted in the vitreous cavity; **(D)** Enucleation was performed and pathological findings revealed no infiltration in the optic nerve head and the sclera. Green Triangle: inner limiting membrane; Red Pentagram: tumor cell cluster; Yellow triangle: damaged photoreceptor cell layer and suspended tumor cells; Red triangle: discrete subretinal tumor cells; Blue triangle: tumor cells are implanted in the vitreous cavity; White pentagram: suspended tumor cell cluster; tumor cell clusters around the optic nerve, subretinal (yellow pentagram) and epiretinal (green pentagram).

A 24-year-old male reported reduced visual acuity in the right eye. Upon examination, his visual acuity was 20/400 in the right eye and 20/20 in the left eye. The condition of the left eye was unremarkable and the anterior segment was normal. Ophthalmoscopic examination of the right eye revealed a circumscribed, nodular, white lesion of the retina located in the peripheral quadrant, coupled with tortuous feeding vessels and diffused subretinal yellow-white deposits (**Figure 2A**). The lesion was 3.2 mm thick and its largest basal diameter was 5.6 mm, as measured by CDI (**Figure 2B**). Swept source-optical coherence tomography angiography (SS-OCT, VG200D, SVision Imaging, Ltd., China, central wavelength: 1050nm; transverse resolution: 15μm [optical]; longitudinal resolution: 5μm [optical]); B-scan revealed that the multifocal subretinal lesions displayed medium- to hyper-reflectivity and exudative retinal detachment involving the macular (**Figure 2C**). Notably, similar punctate lesions with medium to high reflectivity, such as subretinal deposits observed on the lamina cribrosa, as well as around and within the optic nerve, were observed (**Figure 2C**).

Genetic analysis revealed that the RB1 gene variant identified in the patient was a mosaic c.763C>T(p.Arg255Ter) mutation in 1. We performed I-125 plaque brachytherapy, which resulted in significant tumor regression. However, the patient’s condition could not be monitored due to the COVID-19 epidemic. When he returned around 10 months later, we discovered a recurrence of the tumor, which had grown bigger, multifocal, and more diffused than before, as well as fine vitreous seeds overlying the lesion. Enucleation was performed and pathological findings revealed no infiltration in the optic nerve head and the sclera (**Figure 2D**). Furthermore, the subretinal tumor cell clusters shown by pathology corresponded to the multifocal subretinal lesions shown by SS-OCT (**Figure 2C**). At the 18-month follow-up, there was no tumor-related metastasis or death.

### Patient 3

**Figure 3:**
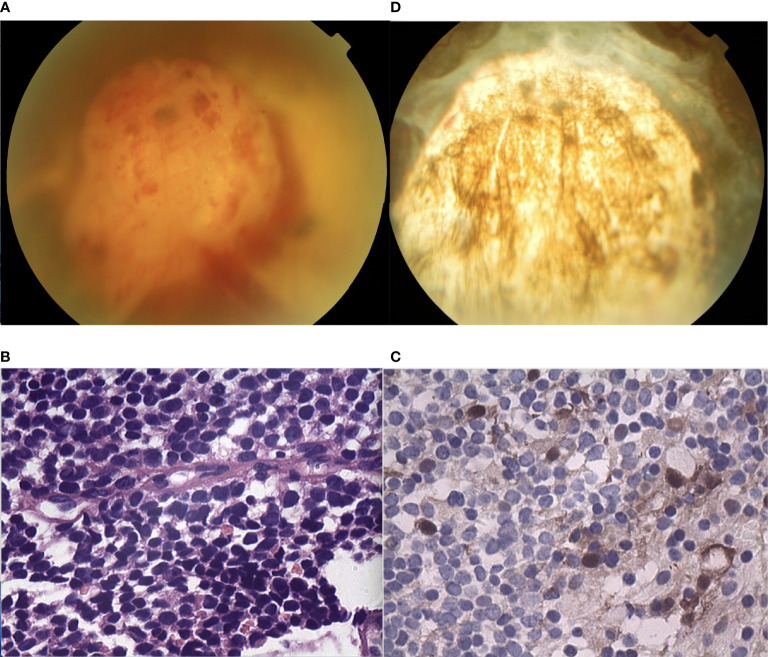
**(A)** In the fundus of the right eye of patient 3, there was a white, dome-shaped lesion of the retina in the inferotemporal fundus, with fine vitreous hemorrhage. The central portion appeared nodular and fibrotic with subtle tortuous retinal vessels. The vitreous seeds were unclear. **(B, C)** Pathology and immunohistochemistry results revealed the foci of **(B)** Flexner-Wintersteiner rosettes that were **(C)** S100 positive. **(D)** In the last follow-up, the retina was well-attached and there was slight proliferation in the defect area of the retina.

A 45-year-old female was found to have a non-pigmented lesion in her right eye during a routine examination. According to the patient, a recurrence of vitreous hemorrhage had occurred twice before the lesion was initially detected two years earlier, but she recovered spontaneously both times. Her visual acuity was 20/20 in each eye. The condition of the left eye was unremarkable. In the fundus of the right eye, there was a white, dome-shaped lesion of the retina with fine vitreous hemorrhage. The vitreous seeds were unclear. The mass was 4.5 mm thick and its largest basal diameter was 6.2 mm. The mass was found in the inferotemporal fundus, and the peripheral portion of the lesion was flat, while the central portion appeared nodular and fibrotic with subtle tortuous retinal vessels, as well as associated radial macular traction (**Figure 3A**). There was a minimally dilated feeding retinal arteriole and draining venules, with associated macular edema, retinal exudation, and subretinal fluid. PET/CT scan was performed to exclude the possibility of metastases. Considering the age of the patient, a benign diagnosis was favorable, and local resection was performed using a 20-gauge vitrectomy for excision of intraocular tumors. The neoplasm was analyzed by pathology and immunohistochemistry. The findings revealed the foci of Flexner-Wintersteiner rosettes, confirming the diagnosis of RB in adults (**Figures 3B, C**). At the 15-year follow-up, the patient’s final visual acuity was 20/100, and there was no evidence of tumor recurrence (**Figure 3D**). Furthermore, there was no tumor-related metastasis or death. The patient declined to undergo genetic testing.

### Patient 4

**Figure 4:**
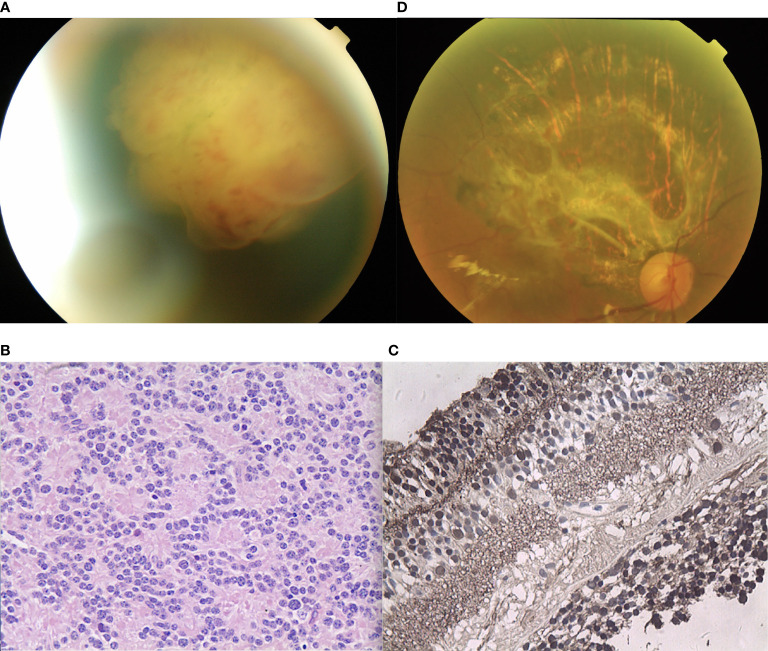
**(A)** The peripheral portion of the lesion in patient 4 was flat and white, while the central portion was elevated. Fine retinal vessels on the surface were observed. **(B, C)** Pathology and immunohistochemistry results revealed the foci of **(B)** Flexner-Wintersteiner rosettes that were **(C)** NSE positive. **(D)** In the last follow-up, the retina was well-attached and there was slight proliferation in the defect area of the retina.

A 55-year-old male experienced mild visual loss over a period of six months in his right eye. Upon examination, his visual acuity was 20/60 in the right eye and 20/20 in the left eye. The condition of the left eye was unremarkable. In the right eye, there was a white, elevated lesion of the retina in the temporal fundus without vitreous seeds. The mass was 3.9 mm thick and its largest basal diameter was 5.8 mm, as measured by CDI. The peripheral portion of the lesion was flat and white, but the central portion was elevated, with fine retinal vessels on the surface (**Figure 4A**). The feeding retinal arteriole and venule were both minimally dilated. There were no associated vitreous seeds, macular edema, or subretinal fluid. Fluorescein angiography revealed that the mass was nonfluorescent with a prominent halo of retinal hyperfluorescence in the late phase. Ultrasonography revealed that the echogenic mass displayed moderate internal reflectivity, suggesting intrinsic vascular pulsations. The patient underwent 18F-FDG PET/CT imaging, which showed no positive uptake in the other parts of the body. The clinical presentation was not consistent with amelanotic choroidal melanoma or metastasis. The patient had local resection using a 23-gauge micro-invasive vitrectomy, and immunohistochemistry results revealed Homer-Wright rosettes and occasional fleurettes, with positive neuron-specific enolase (**Figures 4B, C**). It was then determined that the patient had RB. At the 10-year follow-up, the patient’s final visual acuity was 20/200, and there was no tumor recurrence (**Figure 4D**). Furthermore, there was no tumor-related metastasis or death. Genetic analysis revealed a negative RB1 gene mutation.

### Patient 5

**Figure 5:**
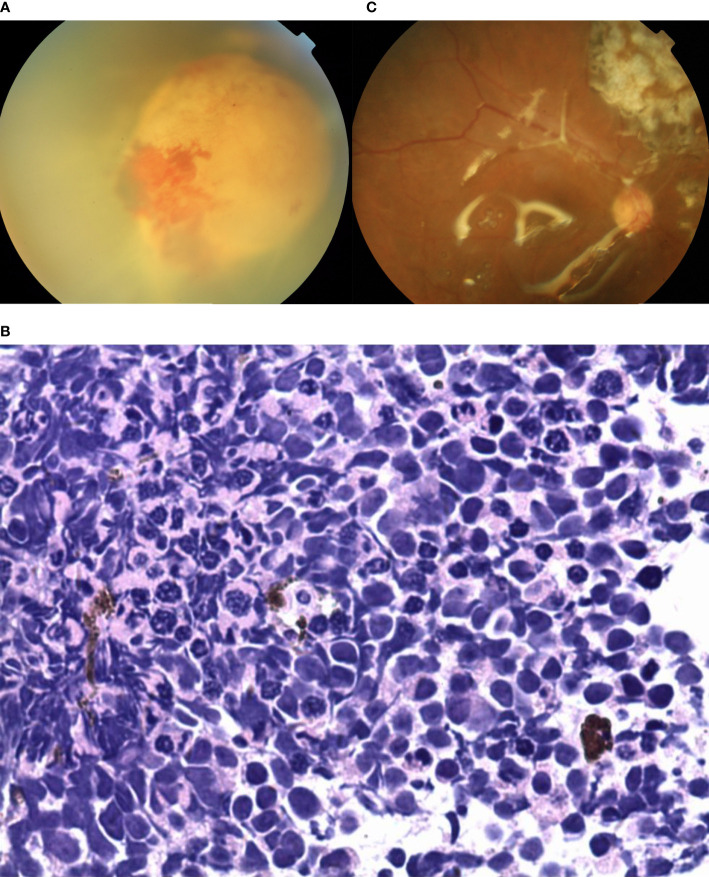
**(A)** In patient 5, there was a white, circumscribed lesion of the retina located in the superotemporal peripheral fundus, with retinal exudation and subretinal fluid in the left fundus. The feeding retinal arteriole and venule of the tumor were minimally dilated and convoluted. There was a mild, focal retinal hemorrhage on the nasal aspect of the lesion with no vitreous seeds. **(B)** Photomicrograph revealed the foci of Flexner-Wintersteiner rosettes. **(C)** In the last follow-up, the retina was well-attached and there was slight proliferation in the defect area of the retina.

A 38-year-old female reported reduced visual acuity in the right eye. Upon examination, her visual acuity was 20/200 in the right eye and 20/20 in the left eye. The condition of the left eye was unremarkable. There was mild, temporal macular traction in the right fundus, while in the left fundus, there was a white, circumscribed lesion of the retina in the superotemporal peripheral fundus, which was associated with retinal exudation and subretinal fluid. The mass was 5.5 mm thick and its largest basal diameter was 6.1 mm, as measured by CDI. The feeding retinal arteriole and venule of the tumor were both minimally dilated and convoluted. There was a mild, focal retinal hemorrhage on the nasal aspect of the lesion, with no vitreous seeds (**Figure 5A**). Fluorescein angiography revealed that the mass was nonfluorescent with a trace of overlying retinal hyperfluorescence in the late frames. Systemic examination revealed no sign of metastases. The patient had local resection using a 20-gauge vitrectomy, and immunohistochemistry results revealed rosette formation and areas of extensive necrosis, but there were no calcific foci (**Figure 5B**). It was then histology confirmed that the patient had RB. At the 6-year follow-up, the patient’s final visual acuity was 20/200, and there was no tumor recurrence (**Figure 5C**). Furthermore, there was no tumor-related metastasis or death. The patient declined to undergo genetic testing.

### Patient 6

**Figure 6:**
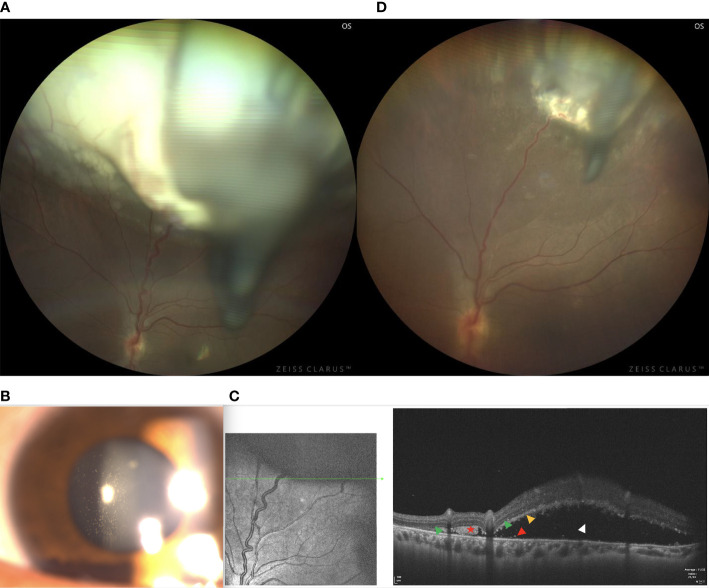
**(A)** In patient 6, there was a white, circumscribed lesion of the retina in the superotemporal peripheral fundus, with tortious seeding vessels, retinal exudation, and subretinal fluid in the left fundus. Significant vitreous seeds were observed. **(B)** The slit lamp microscope showed significant tumor seeds floating in the vitreous cavity. **(C)** The SS-OCT B-scan revealed local exudative retinal detachment surrounding the tumor, as well as subretinal deposition spots with medium to high reflectivity without involving the macular. (Green Triangle: outer limiting membrane; Red Pentagram: tumor cell cluster; Yellow triangle: damaged photoreceptor cell layer and suspended tumor cells; Red triangle: discrete subretinal tumor cells; Blue triangle: tumor cells are implanted in the vitreous cavity; White triangle: superficial retinal detachment) **(D)** Three months after I-125 plaque brachytherapy, the tumor showed a reduction in size, resolution of subretinal fluid, normalization of the caliber and decrease in the tortuosity of feeding and draining vessels.

A 24-year-old female reported floaters in the left eye. Upon examination, her visual acuity was 20/60 in the right eye and 20/25 in the left eye. The condition of the right eye was unremarkable. In the left fundus, there was a white, circumscribed lesion of the retina in the superotemporal peripheral fundus with tortious seeding vessels, retinal exudation, and subretinal fluid (**Figure 6A**) The feeding retinal arteriole and venule of tumor were both minimally dilated and convoluted, with significant vitreous seeds (**Figure 6B**). The mass was 4.8 mm thick and its largest basal diameter was 13.7 mm, as measured by CDI. SS-OCT B-scan revealed a local exudative retinal detachment surrounding the tumor, as well as subretinal deposition spots with medium to high reflectivity, not involving the macular (**Figure 6C**). Genetic analysis revealed a negative RB1 gene mutation.

Vitreous biopsy was then performed, followed by pathological analysis. The findings revealed the foci of Flexner-Wintersteiner rosettes, confirming the diagnosis of RB in the adult. We then performed I-125 plaque brachytherapy, which resulted in tumor reduction 3 months later (**Figure 6D**) and stable visual acuity. However, there were still fine white vitreous seeds overlying the lesion. Therefore, a further intravitreal injection of melphalan or topotecan was required. Systemic examination revealed no signs of metastases.

## Discussion

The possibilities in the diagnosis of an amelanotic mass lesion of the fundus in an adult include amelanotic melanoma, lymphoma, metastatic carcinoma, astrocytoma, tubercular choroiditis, endophthalmitis, inflammatory diseases of the retina, RB, and retinoma (4, 7–9).

The diagnosis of RB should be considered in adults with an amelanotic whitish mass lesion (pseudoretinoblastoma) (10, 11) in the fundus. However, it remains a challenge due to its rarity. Almost all RBs in adults are sporadic and unilateral. In our six cases, adult-onset RB displayed unique clinical characteristic signs on fundus appearance, including 1) unilateral diseases, 2) white mass originating from the retina with vitreous seeds, 3) tumor-associated feeding vessels, exudative retinal detachment, 4) sub-retinal discrete white-yellow deposits (tumor cells clusters), 5) uncommon calcification, and 6) rare tumor-related metastasis or death. Therefore, lesions that simulate a true retinal capillary hemangioma, as well as choroidal osteoma with retinal invasion can be mistaken for an RB lesion.

Although large white lesions with vitreous seedings can easily be identified as RB, accurate diagnosis of smaller lesions and early lesions is an issue. Ultrasonography and CT scan can reveal calcification and characteristic imaging patterns. However, in certain cases, the diagnosis may be unclear even when both approaches are used. In all our cases, both imaging methods did not detect any calcification. Fine-needle aspiration cytology and immunohistochemistry (with neuron-specific enolase) may help confirm a diagnosis, but the former approach is controversial due to the risk of tumor cell dissemination (12). SS-OCT revealed multifocal, punctate, or spot subretinal lesions with medium- to hyper-reflectivity involving the lamina cribrosa and the surrounding of the optic nerve (**Figures 2C, D**), as well as the spot lesions corresponding to subretinal tumor cells in pathological findings (**Figures 2C, D**). These tumor cell clusters could be exfoliated, inactive tumor cells from the original mass; however, based on the theory that RB originates from ARR3-positive maturing photoreceptor precursors cells (13), these tumor cell clusters could be early RB lesions gathered by active tumor cells, which may be related to metastasis and a worse prognosis. As pathological analysis of enucleation (patient 2) showed no evidence of invasion in the optic nerve, which was not consistent with the SS-OCT findings (tumor celon the surface of the lamina cribrosa), we speculated that this may be due to the differences in the scanning direction and resolution between SS-OCT (transverse resolution: 15μm [optical]; longitudinal resolution: 5μm [optical]) and the pathological section (4 μm). Therefore, tumor invasion of the lamina cribrosa and the optic nerve should be interpreted with caution, and this patient is still being closely monitored.

Tumor histopathology or enucleation confirms the diagnosis and identifies tumor differentiation. Among our cases, patients 3 and 4 had well-differentiated RBs, while another two had undifferentiated tumors. For patient 2, enucleation pathology showed that the optic nerve head and the sclera were not infiltrated. Tumors with rosettes typically have a central lumen surrounded by a single row of cells with scanty cytoplasm and large oval nuclei with nucleoli. Nuclear pleomorphism and mitotic activity are commonly observed in these cells. Tumors displaying foci of Flexner-Wintersteiner rosettes are classified as differentiated RB.

RB, a rapidly growing tumor derived from embryonal retinal cells, is usually caused by biallelic loss of the RB1 gene, a tumor suppressor gene at chromosome 13q14, during infancy and childhood (14). RB in adults is rare (7, 9), and genetic studies on adult-onset RB patients are limited. The cause of RB in adults was speculated to be the reactivation of previously undiagnosed, spontaneously regressed, or arrested RB (also termed retinocytoma) (2). In this study, patients 1 and 2 had the RB1 mosaic mutation. According to a recent study, the proportion of low-level deleterious copy number variant mosaicism in the blood is over 4% and low-level mosaicism is considered an under-recognized cause of disease (15). Notably, the variant of mosaic c.709dupG (p.Glu237GlyfsTer4) duplication in patient 1 had not been previously reported but was expected to cause RB due to a frameshift and premature stop codon, resulting in unstable mRNA transcript or truncated protein. Therefore, the relevant pathogenic gene was identified in adult-onset RB cases, and it is important to note the genetic differences between childhood and adult-onset RB.

Undetected RB1 mutations in fully tested tumors (RB1+/+) may include deep intronic mutations, translocations, or alterations in unknown RB1 regulatory regions. Certain unilateral RBs with undetectable RB1 mutations arise *via* an independent mechanism. Rushlow (16) and colleagues reported that there were no RB1 mutations (RB1+/+) in approximately 2.7% of unilateral, non-familial children with RB tumors. Furthermore, they identified a distinct RB1+/+MYCNA subtype that has no RB1 mutations, displays functional protein, and accounted for 1.4% of the 1068 samples. In this study, there were insufficient RB1+/+ tumor samples for gene expression or protein analysis. There was no RB 1 mutation in two patient blood samples, while another two patients had the mosaic RB 1 mutation. These findings suggested that the prognosis of adult-onset RB is favorable and merits further investigation.

The management of adult-onset RB is determined by the stage of the disease and conditions of therapy at the time of manifestation. At present, globe preserving treatment remains a challenge for eyes with adult-onset RB. Enucleation was the primary treatment modality in most reported cases, as the lesions were detected at a fairly advanced stage and each patient had one normal eye. According to previous studies on adults with RB, the disease is usually treated with excision or enucleation at an advanced stage (Group D or E) (7, 9, 17). However, some vision in the eyes of certain unilaterally affected patients may be saved using inexpensive and non-invasive treatment. The selection criteria and treatment guidelines we followed were based on previous reports and experience with our patients. In this study, the TTT, IVM, I-125 plaque brachytherapy, surgical resection, and combination therapy were used as the primary local treatments for adult-onset RB, with none of the patients developing metastasis or died until the final follow up. In addition, because intraarterial chemotherapy is an invasive procedure and there was no indication that systemic chemotherapy was needed, we did not use chemotherapy (either intraarterial or intravenous) as the primary treatment. Similar to two of our patients, a few patients from previous studies underwent I-125 plaque brachytherapy. Although the curative effect appeared to be good for tumor regression in the early stages, there was tumor recurrence and poor results at diagnosis and follow-up in the advanced stages of the disease. Three patients underwent local tumor resection but did not experience tumor recurrence or metastasis. The optimal treatment for adult RB should still be evaluated with caution due to the rarity of the disease.

Since RB in adults is extremely rare and there are no established treatment protocols, we referred to the treatment principles of children with RB. Melphalan is the most extensively used drug to control the vitreous seeds in RB (18–22). However, Francis et al. (21, 23) discovered that melphalan injection caused a decrease in ERG response, leading them to speculate that more deeply pigmented eyes absorb increased levels of melphalan, resulting in enhanced RPE toxicity, and, by extension, retinal and choroidal toxicity. When patient 1 was administered intravitreal injection of melphalan for Rb vitreous seeds, there was obvious drug toxicity despite its effectiveness. Therefore, the dosage or results of IVM in adult-onset RB must be interpreted with caution. Shields et al. used topotecan for vitreous seeds in RB as it was cheaper, less toxic, and effective (24–26). Melphalan is no longer used by a group from India due to its toxicity. Although some studies reported that topotecan is less effective than melphalan, Rao et al. (26) found it to be very effective. Therefore, we may use 20 ug/0.1 cc topotecan in the future for refractory or recurrent vitreous seeds in RB.

In summary, we reported a series of cases of RB in adults, describing their clinical characteristics and rare genetic make-up, as well as outcomes of local management as primary therapy. In the presence of amelanotic whitish mass lesions in the fundus of an adult, the possibility of RB as a clinical diagnosis should be taken into consideration. If the diagnosis is still unclear, RB1 gene testing may be recommended. SS-OCT can be recommended as the primary investigation method since it can detect fine lesions and shed light on the development and therapeutics of RB. Although adult patients with RB have historically poor globe salvage rates, early diagnosis of the disease and appropriate treatments improve globe salvage and long-term survival of adults with RB compared to children with RB. Based on our findings and the literature, patient survival was minimally affected, with no tumor-related metastasis or death.

## Data Availability Statement

The raw data supporting the conclusions of this article will be made available by the authors, without undue reservation.

## Ethics Statement

The studies involving human participants were reviewed and approved by Institutional Review Board of Beijing Tongren Hospital. The patients/participants provided their written informed consent to participate in this study.

## Author Contributions

WW: Examination of the patient, interpretation of results, writing the manuscript; NZ: Interpretation of results and writing/reviewing of the manuscript; XX: Interpretation of results and reviewing of the manuscript. LY: Reviewing and examination of the manuscript. YL: Examination and treatment of the patient. All authors read and approved the final manuscript.

## Funding

The National Natural Science Foundation of China (Nr. 81272981), the Beijing Natural Science Foundation (Nr. 7151003) provided financial support.

## Conflict of Interest

The authors declare that the research was conducted in the absence of any commercial or financial relationships that could be construed as a potential conflict of interest.

## Publisher’s Note

All claims expressed in this article are solely those of the authors and do not necessarily represent those of their affiliated organizations, or those of the publisher, the editors and the reviewers. Any product that may be evaluated in this article, or claim that may be made by its manufacturer, is not guaranteed or endorsed by the publisher.
